# Intraoral Epithelioid Hemangioendothelioma: an Intermediate Vascular Tumor- A Case Report

**Published:** 2009

**Authors:** Bhari Sharanesha Manjunatha, Gopal Siva Kumar, Raghunath Vandana

**Affiliations:** * Reader in Oral and Maxillofacial Pathology, KM Shah Dental College and Hospital, Waghodia, Vadodara, Gujarat, India; ** Dean And Head, Department of Oral and Maxillofacial Pathology, K.S.R. Institute of Dental Sciences and Research, K.S.R. Kalvi Nagar, Tamil Nadu, India; *** Post Professor and Head, Department of Oral and Maxillofacial Pathology, Narayana Institute of Dental Sciences, Nellore, Andhra Pradesh, India

**Keywords:** Epithelioid, Hemangioendothelioma, Hemangiosarcoma

## Abstract

Vascular neoplasms, other than benign are characterized as intermediate or malignant. They are often enshrouded in controversy, because the same neoplasm could show variability in biologic behavior that may not be correlated with microscopic features. The intermediate grade vascular neoplasm is named as epithelioid hemangioendothelioma (EHE). Epithelioid hemangioendothelioma of the oral cavity has been infrequently reported. To the best of our knowledge, the review of the English literature revealed a total of 30 cases of intraoral EHE reported till today. We report such a rare case in a 20 year old male, presented with a growth in lower anterior lingual gingiva since five months before the diagnosis with a history of similar swelling, twice in the same area. The differential diagnosis and brief review of literature is also discussed in the current article.

## Introduction

Epithelioid hemangioendothelioma (EHE) is considered as an intermediate vascular neoplasm between hemangioma and angiosarcoma. It is characterized by neoplastic proliferation of epithelioid/histiocytoid endothelial cells.^1^ Tumors of intermediate behavior have the ability to recur locally and metastasize, but at a far reduced level compared to malignant ones.^2^

The behavior of oral EHE is not predictable due to lack of agreement in terminology, criteria for diagnosis and difference in biologic behavior relative to its anatomic site and age of occurrence.^3^ Clinically, oral EHE strongly mimics benign reactive lesions such as pyogenic granuloma, chronic periodontal disease, peripheral giant cell granuloma and patients usually present with ‘bleeding soft tissue mass and resemble friable granulation tissue’ in as many as 75% of cases.^3^

EHE of the oral cavity has been infrequently reported. To the best of our knowledge, the review of the English literature revealed a total of 30 cases of intraoral EHE reported till today.^4^–^9^ A detailed review by Chi et al^6^ in 2005 suggested only 14 cases of intraoral EHE, including one intraosseous in the posterior mandible and second case seen as gingival nodule. On the contrary, Anderson et al^10^ in 2003 have reported EHE of the lip in an 18 year old woman.

Uehara et al^7^ in 2006 counted 15 cases and added their case of EHE of tongue in a 72 year man. Very recent reports^8^,^9^,^11^ suggest a total of 30 cases in the world of language English literature till the end of 2008.

According to Chi et al^6^ the average age was 28 years with a range of 4 to 76 years and a female-to-male ratio of 2.5:1. Gingiva or alveolar mucosa was the most commonly affected intraoral locations. Most lesions appeared erythematous, purple pink to yellowish in color and were asymptomatic.

However, reports by Uehara et al,^7^ Mohtasham et al^9^ and Naqvi et al^11^ showed the average age of 46.8 years with a range of 9 to 72 years and a male predilection. Again gingiva was the most common area of occurrence. A total of 5 cases were reported by these authors. On the other hand, these cases do contribute for variations in epidemiological figures. A recent review^8^ comprised of seven males and two females aged 6-53 years with a mean age of 28 years. The predominant site of the tumor included the tongue in about 4 cases.

Our case presented as a painless gingival growth in lower lingual anterior area causing difficulty in speech. The lesion had recurred twice and was treated by local excision of the soft tissue growth. No signs of metastasis to either regional lymph nodes or any other organ was noted. Because of its rarity, a case of EHE of the oral cavity is reported.

## Case Report

**Figure 1:**
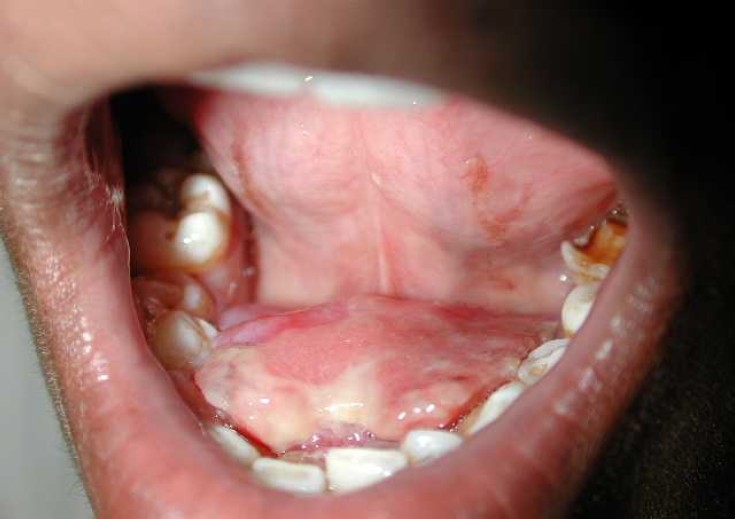
Asymptomatic, erythematous mass on the lower lingual gingiva in the midline

A 20 years old male patient hailing from a sub-urban area was referred to the hospital with a complaint of difficulty in speech due to a growth in lower anterior lingual area since five months ago. The mass was asymptomatic, pedunculated and measured about 3 × 4 centimeters seen on the lingual aspect of lower anterior gingiva in the midline (Figure 1).

The surface of the lesion was rough, non-tender with soft to firm in consistency and was erythematous. He had the habit of smoking for 2 years and 5-6 times/day. Grade I mobility of all permanent incisors (bilateral) in the same area was noted. Complete blood examination was also done which was non-contributory.

**Figure 2:**
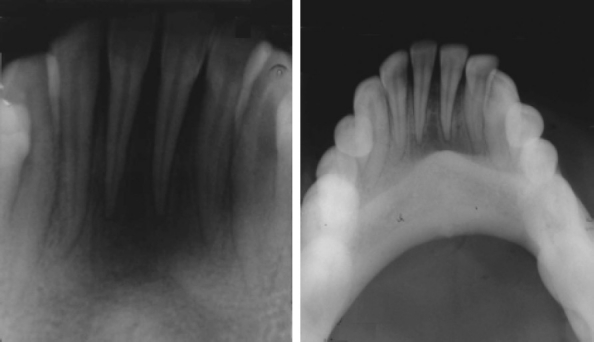
IOPA and Occlusal radiographs showing saucer like bony erosion of underlying alveolar bone in the midline extending bilaterally up to canines.

On questioning, he gave a history of similar lesions in the same area. First time, the growth was of gradual onset and progressive in nature occurred back in 8 years. In the second time (first recurrence), the lesion was noted 1 year back. In both occasions, he was treated by a local general dentist and the histopathological reports were similarly suggestive of ‘nonepithelial small cell round tumor’. Considering the history and histopathological report of previous lesion, IOPA and occlusal radiographs were advised. Radiographic examina-tion showed erosion of underlying alveolar bone (Figure 2).

A provisional diagnosis of ‘peripheral giant cell granuloma’ was made and an incisional biopsy was done.

**Figure 3:**
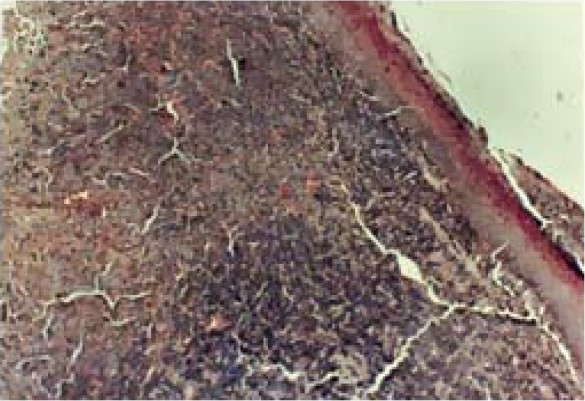
Photomicrograph showing sub-mucosal nodule of tumor mass arranged in clusters/nests of cells close to overlying oral epithelium (hematoxylin and eosin staining × 100).

Microscopically, the lesion showed round to ovoid cells arranged in clusters/nests seen close to atrophic epithelium (Figure 3).

**Figure 4:**
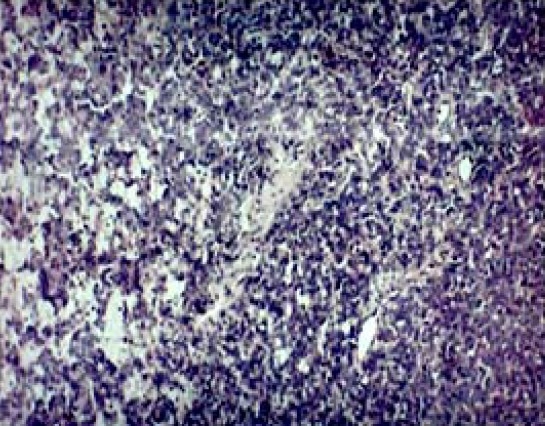
Photomicrograph showing hyperchromatic to vesicular nuclei close to small and medium sized blood vessels in a myxoid to scanty collagenised stroma (hematoxylin and eosin staining × 250)

The tumor cells were large mimicking epithelial cells having hyperchromatic to vesicular nuclei with few mitotic figures. Closely associated blood vessels in a myxohyaline to scanty collagenised stroma were noted (Figure 4).

**Figure 5:**
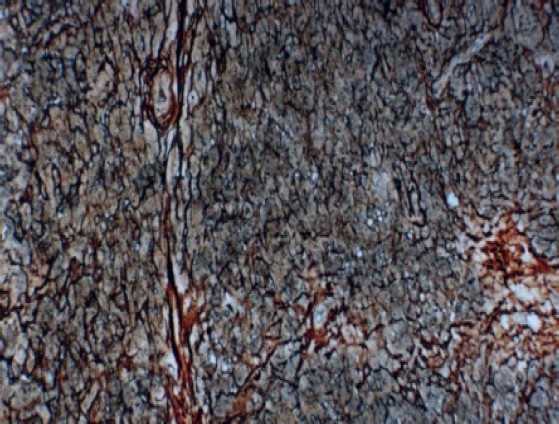
Photomicrograph showing epithelioid cells with intracytoplasmic lumen formation and few mitotic figures (hematoxylin and eosin staining × 250)

**Figure 6:**
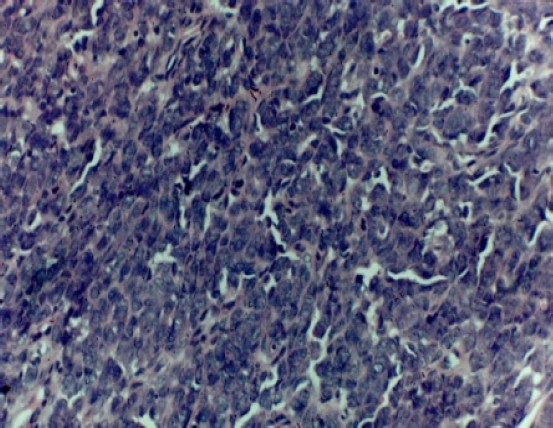
Photomicrograph showing epithelioid tumor cells having vacuolated cytoplasm with lumen and also shows nuclear and cellular pleomorphism (hematoxylin and eosin staining × 400).

Interestingly, epithelioid cells had vacuolated cytoplasm with lumen formation and also showed nuclear and cellular pleomorphism (Figures 5 and 6).

**Figure 7:**
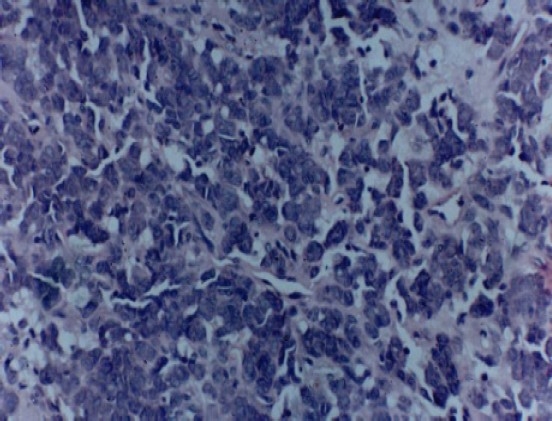
Photomicrograph showing dense reticulin network surrounding tumor cells, confirming the tumor origin from endothelial cell (reticulin staining X 250).

Mucicarmine staining was done and was negative for mucin formation. Later, reticulin staining was performed, which showed a dense network of fibers around the tumor cells (Figure 7), confirming the endothelial cell origin and the final diagnosis of ‘epithelioid hemangioendothelioma’ was made.

The patient was advised for complete surgical excision of the lesion and unfortunately, no further treatment was done as we lost the follow-up of patient.

## Discussion

The term hemangioendothelioma (HE) was introduced by Borrmann in 1899 who proposed it as an intermediate or low grade malignant potential vascular neoplasm. EHE is a very uncommon vascular neoplasm, first described by Enzinger and Weiss in 1982.^1^ It is seen mainly in the soft tissues of extremities; but can also be seen within the bone and other parts of the body. EHE occurs rarely in head and neck region and was reported in the oral cavity by Ellis and Kratochvil in 1986.^12^

The clinical differential diagnoses when seen on the gingival area include pyogenic granuloma, peripheral giant cell granuloma and periodontitis.^3^

No consistent clinical or histologic criteria for predicting the biologic behavior of EHE in the oral region have yet been identified.

The microscopic differential diagnosis includes metastatic carcinoma, adenocarcinoma, epithelioid angiosarcoma and melanoma.^1^,^2^

Lack of higher degrees of nuclear atypia and mitotic figures ruled out epithelial malignancies such as metastatic carcinoma and melanoma. Also, these lesions fail to stain with reticulin stains.^1^–^3^ Epithelioid angiosarcoma usually shows solid nests of endothelial cells with high mitotic activity along with necrosis, a common finding in such lesions.^5^

Hemangioendothelioma can imitate hemangiopericytoma in routine hematoxylin and eosin stain, but silver reticulin stain can differentiate these two lesions.^13^ When the tumor cells are intraluminal or lie inside the reticulin network that encloses the vessel, they are of endothelial origin. On the other hand cells located outside the network are of pericyte origin.^3^,^7^,^14^

## Conclusion

A case of epithelioid hemangioendothelioma of lower lingual gingiva was described here. The literature regarding intraoral epithelioid hemangioendothelioma was reviewed from the available world English literature. Only 30 cases have been reported till 2008. In many cases, the common clinical picture was a benign lesion in a middle aged patient located on gingiva.

We conclude that though intraoral epithelioid hemangioendothelioma is rare, the clinician should be aware of clinical and histopathological features of this lesion. More importantly, a dental practitioner should always send any tissue taken from oral cavity for histological examination as part of treatment of commonly lesions which mimic reactive lesions such as pyogenic granuloma, giant cell granuloma, fibroma, mucocele and others. Epithelioid hemangioendothelioma may turn into malignancy or recur or may metastasize to regional lymph nodes. If diagnosed as epithelioid hemangioendothelioma, complete surgical excision with wide margins with long term follow up is highly recommended.
